# A Second-Generation Device for Automated Training and Quantitative Behavior Analyses of Molecularly-Tractable Model Organisms

**DOI:** 10.1371/journal.pone.0014370

**Published:** 2010-12-17

**Authors:** Douglas Blackiston, Tal Shomrat, Cindy L. Nicolas, Christopher Granata, Michael Levin

**Affiliations:** 1 Biology Department and Center for Regenerative and Developmental Biology, Tufts University, Medford, Massachusetts, United States of America; 2 Department of Regenerative and Developmental Biology, Forsyth Institute, Boston, Massachusetts, United States of America; 3 Boston Engineering Corporation, Waltham, Massachusetts, United States of America; University of Queensland, Australia

## Abstract

A deep understanding of cognitive processes requires functional, quantitative analyses of the steps leading from genetics and the development of nervous system structure to behavior. Molecularly-tractable model systems such as *Xenopus laevis* and planaria offer an unprecedented opportunity to dissect the mechanisms determining the complex structure of the brain and CNS. A standardized platform that facilitated quantitative analysis of behavior would make a significant impact on evolutionary ethology, neuropharmacology, and cognitive science. While some animal tracking systems exist, the available systems do not allow automated training (feedback to individual subjects in real time, which is necessary for operant conditioning assays). The lack of standardization in the field, and the numerous technical challenges that face the development of a versatile system with the necessary capabilities, comprise a significant barrier keeping molecular developmental biology labs from integrating behavior analysis endpoints into their pharmacological and genetic perturbations. Here we report the development of a second-generation system that is a highly flexible, powerful machine vision and environmental control platform. In order to enable multidisciplinary studies aimed at understanding the roles of genes in brain function and behavior, and aid other laboratories that do not have the facilities to undergo complex engineering development, we describe the device and the problems that it overcomes. We also present sample data using frog tadpoles and flatworms to illustrate its use. Having solved significant engineering challenges in its construction, the resulting design is a relatively inexpensive instrument of wide relevance for several fields, and will accelerate interdisciplinary discovery in pharmacology, neurobiology, regenerative medicine, and cognitive science.

## Introduction

### Automated behavior analysis

Fundamental understanding of the mechanisms of cognition, behavior, and memory require the synthesis of insights from genetics, developmental neuroscience, and ethology in molecularly-tractable model organisms. While some groups are beginning to add analyses of cognitive endpoints to their pharmacological and genetic perturbations [1], [2], [3], [4], a significant barrier prevents the ready investigation of the causal chain leading from genetics, through the embryonic establishment of CNS structure, to behavior. Similarly, developing the next generation of nootropic drugs (e.g., for enhancement of intelligence or memory) requires the introduction of high-throughput screening technologies beyond single-cell *in vitro* systems or automated detection of behavioral defects.

Performing behavior experiments in a fully automated, computer-controlled, quantitative platform has several advantages. First, bias and experimenter effects inherent in manual procedures are minimized, allowing more consistent, quantitative, rigorous behavioral analyses [5]. Second, it becomes feasible to train and quantify the behavior of large numbers of individuals simultaneously. Third, automation allows for essential control conditions such as yoked controls, in which control animals receive rewards and punishments based on the behavior of other animals being trained, and ensures that consistent training protocols can be maintained for as long as needed (e.g., continually across multiple days, ensuring more robust learning). Fourth, it makes it possible for investigators at distant locations to reexamine experiments using identical training protocols in similar environment, increasing the cross-compatibility of datasets and validity of analyses.

### Current state of the art – advances and limitations

The difficulties of ensuring accessible, standardized, quantitative behavior analysis in labs specializing in molecular genetics or developmental neurobiology is a significant barrier to progress in many fields. While automated analysis of specific metrics in mouse behavior is now routine [6], [7], [8], [9], [10], [11], [12], some of the most exciting progress in linking cell dynamics to cognitive functions are coming from systems such as zebrafish, *Drosophila*, *C. elegans*, and *Xenopus laevis*. A few systems have been developed for small species that are amenable to screening and large population studies such as crustaceans [13], zebrafish [14], [15], [16], *Drosophila* [17], *C. elegans* [18], [19], and planaria [20], [21], [22], [23], [24], [25], [26]. The development of unique, highly-specialized devices has had limited impact on labs without expertise in engineering, access to an electronics facility, and programming knowledge, since reproduction and adaptation of an existing system from another group can present formidable challenges.

Several commercial efforts have attempted to fill the gap. The most popular off-the-shelf solutions for automated tracking systems include Ethovision (Noldus Information Technology) and Videotrack/Zebralab (Viewpoint Life Sciences). Both systems offer automated behavior analysis from video feeds or stored video files and can track multiple organisms, typically from a single camera imaging a number of individual dishes or 96 well plates. Viewpoint software has been used to study circadian systems and sleep [27], [28] as well as neuroactive drugs and addiction [29], [30] in zebrafish. Software from Noldus has been used both molecularly, to study alcohol effects and anti epileptic drugs [31], [32], [33], as well as ecologically to examine how fish respond to allopatric/sympatric/animated predators, conspecifics, or alarm substances [34], [35], [36], [37] and has produced similar results to standard manual recording procedures [38].

A variety of custom software has also been developed for specific tasks, including tracking multiple organisms that cross paths or following individuals in three-dimensional space [39], [40]. However, all existing commercially-available systems lack a key feature: the ability to perform automated training and learning tasks in real time. To address a more complete repertoire of questions about behavior (as well as perform screens of mutations or drugs that affect learning and memory), it is necessary to be able to perform operant conditioning as well as simpler associative learning. Operant (instrumental) conditioning requires that a system not only be able to track multiple individuals in parallel but must also give immediate individual feedback to each organism based on behavior. For example, if lights or shock are used as training stimuli in a task where an animal is taught to avoid a moving light or stay in a particular region of the dish, they must be executed in real time in response to the potentially different actions of each individual, and the stimuli must be insulated from all surrounding members undergoing training. Table 1 lists the rich variety of behavioral paradigms and illustrates which are and are not possible using current commercial systems.

**Table 1:** Trial types possible in this system.

Quantitatively characterize average movement rates (acceleration, activity rate)
Quantitatively characterize types of motion (does it move in circles? Does it move in straight lines of specific size before changing direction?)
Quantitatively characterize curiosity (tendency for dish exploration)
Quantitatively characterize response to lights flickering at a specific rate
Quantitatively characterize the variability of behavior of individual animals from the same mother, or from different mothers, with respect to: thresholds of sensitivity to sound and light stimuli, types of responses to sound and light stimuli, preferred location in dish and motion types, and rate and acceleration of movement
Quantitatively characterize response to light of different brightness levels
Quantitatively characterize propensity to stay near the edge of the dish
Quantitatively characterize propensity to move in specific types of patterns
Quantitatively characterize circadian rhythms (variability of behavior as a function of daytime)
Quantitatively characterize responses to light stimuli presented to different parts of the animal (from behind, to the left, to the right)
Quantitatively characterize responses to electromagnetic fields in the environment
Quantitatively characterize response to electric shocks
Quantitatively demonstrate instrumental or classical conditioning; for example, individually train the animals to do any of the following: stay at the dish edge; stay in the center of the dish; move every time a particular vibration occurs; freeze when vibration stops; follow the lit-up quadrant as it moves; stay in the dark; keep moving at a certain rate; swim clockwise when light flickers at a certain rate
Quantitatively assay effects of compounds or mutations on learning rate
Quantitatively assay effects of compounds or mutations on retention of learned information

While salesmen generally tell customers that, for example, “shock effectors could easily be added” to their system, the reality is that numerous engineering, fabrication, and software issues must be overcome in order to adapt an existing tracking system and enable it to give positive and negative feedback to each animal in real time. Moreover, the various requirements are highly interdependent, and multiple cost-feature tradeoffs have to be managed. It is difficult for an investigator who wants to perform behavioral experiments to test numerous choices of cameras, lenses, electrodes, and other components. Creating individual animal chambers (amenable to differential light levels and spatially-homogenous shock with no “special points” due to electrodes breaking radial symmetry of the dish) and modifying software to control them at high speeds in real time is a very difficult task.

### A second-generation, automated training and analysis system

Our group sought to investigate the effects of duplication of brain and CNS structures on behavior [41], [42]. Key requirements for such research programs include the ability to: (1) characterize multiple animals in any one run (for statistical power despite individual variability), (2) provide differential environmental cues and feedback (reward/punishment) to individual animals in real time (enable analysis of memory and learning, which proceeds differently for each animal), (3) gather separate behavior data timelines on each animal (4) afford a high level of automation and rich data acquisition (to provide comprehensive analysis while minimizing experimenter effects and optimizing efficiency and reproducibility of protocols among labs), (5) be usable with several model organisms, (6) allow a large parameter space of possible training paradigms and stimuli covering several different sensory modalities (flexibility), and (7) acquire and use the system rapidly, without a long period of expensive custom modification by each lab (affordability and turn-key operation). Since no satisfactory system could be commercially obtained, we addressed these challenges to produce a system [43] with the following properties.

The platform was to contain individual “Skinner chambers” sized to fit standard Petri dishes, within which single animals could be provided with light and mild electric shock stimuli. It must allow for the programming of individualized combinations of environmental cues and feedback (reward/punishment) in real time to accommodate different rates of memory and learning among animals within a sample group. The system should be modular, flexible, convenient, and powerful, allowing use by operators without computer programming skills, while using off-the-shelf components and costing end-users no more than a mid-range fluorescence microscope system. Its design should be flexible enough for the convenient inclusion of essential control conditions such as yoked controls, in which control animals receive rewards and punishments based on the behavior of other animals being trained. Primary data must be recorded over long time periods and be available for subsequent review, analysis and transfer to other laboratories. It was important to incorporate scaleability to increase throughput, expandability to include other model organisms and additional assays (e.g., maze studies), and ease of use and maintenance. Our prototype was tested in two species specifically chosen for their wide-spread use in several fields, amenability to molecular-genetic, developmental, pharmacological, and behavioral analyses, and different physical and optical properties (to maximally stretch the applicability and flexibility of the system).

### Planaria as a model system for behavior

Free-living flatworms, planarians, can regenerate a whole worm from only a small section of the adult [42], [44], [45]. Besides regenerative biology, they are also a popular system for the study of cancer [46], [47], the neuropharmacology of drug addiction [48], [49], [50], [51], [52], [53], and memory and learning [23], [54], [55], [56], [57], [58], [59], [60]. They are a critical breakthrough in the evolution of the animal body plan, representing the “first” organism to have both bilateral symmetry and a centralized brain with true synaptic transmission [61]. Planarians exhibit much of the complexity of vertebrate systems, including a well-differentiated nervous system (with most of the same neurotransmitters as human brains), eyes, brain, three body layers, and bilateral symmetry. Thus, planaria are a unique model organism for studying the mechanisms that underlie the regeneration of a functional nervous system and the restoration of cognitive structures after injury by adult stem cells.

### Aquatic vertebrates as a model systems for behavior

The tadpoles of the African clawed frog *Xenopus* possess a complex CNS and exhibit rich social behaviors (schooling), kin recognition, and the ability to modulate growth rate based on visual appraisal of conspecific density [62], [63]. While some reports have noted difficulty when training tadpoles manually [64], a number of species have been shown to learn efficiently in simple paradigms using electric shock [65], [66], [67], [68], [69], [70], [71], vibrations [72], [73], [74], odor avoidance [75], and conditioned place preference [76]. Molecular manipulations currently allow the study of *Xenopus* larvae with extra eyes [77], [78], altered brain compartment sizing along anterior-posterior and medio-lateral axes [79], [80], [81], reversed laterality [82], [83], multiple brains [41], [84], [85], hyper-innervated muscles, and altered complements of neurotransmitter receptors throughout the CNS [86], [87], [88]. Given their sensory and behavioral repertoires, learning capabilities, and prominent role as a model system in developmental biology, *Xenopus* larvae are an excellent subject for systems biology approaches to cognitive function. An automated system, increasing both the statistical power and the number of different kinds of assays to be tested, will be essential to establishing a paradigm to ask in a rigorous and quantitative way how the brain of various model species handles alterations in somatic and sensory organs, as well as changes in nervous system structure.

Comparatively more is known about behavior, learning, and memory in the zebrafish *Danio rario*. Fry and adult fish have been studied in a variety of ecological contexts including shoaling, predation, feeding, mate choice, and social transmission of information through release of alarm pheromone [34], [35], [36], [37], [89], [90], [91], [92], [93]. In addition, this organism is positioned at the forefront of molecular inquiries in the field of human health given its rich history of forward and reverse genetic approaches, as well as its amenability to pharmaceutical screening in 96-well plates [31], [32], [94], [95]. Thus, zebafish are a well-established model system for investigating the basis of of addiction, memory consolidation, eye disease, and sleep [27], [28], [32], [33], [94], [96], [97], [98], [99], [100], [101], [102]. Combining the ability to generate hundreds of mutant animals with a device capable of automating learning and memory assays is a particularly attractive match; it has the potential to generate huge amounts of data on the genetic mechanisms of normal cognitive phenomena as well as disorders. In addition, comparing data between frog and fish (within the same apparatus under identical conditions) offers a powerful “evo-devo” perspective in which conservation and divergence of results between vertebrates can be studied.

We built a system that can be readily applied to studies in *Xenopus*, planaria, zebrafish, and similar model organisms in any laboratory. Here we describe this platform and present sample data illustrating its use.

## Methods

### Physical Device

**Figure 1:**
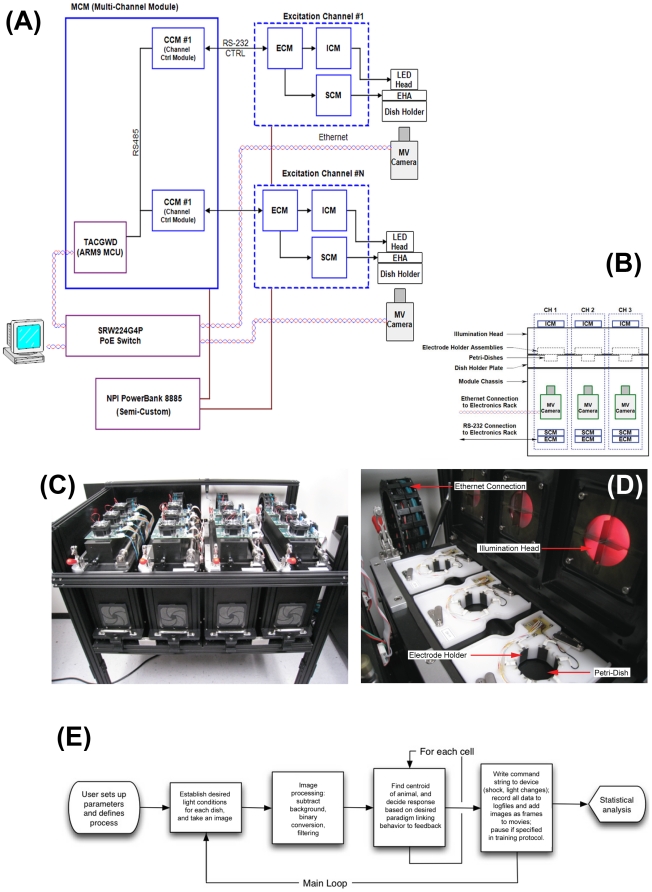
Schematic diagram and physical construction of the automated behavior device. The system consists of a bank of individual cells connected to controllers, with the whole device running an embedded Linux system and communicating with a master PC computer via Ethernet networking. Panel (**A**) shows the top-level system architecture. The components are as follows. Training Apparatus Controller Gateway Device (TACGWD) and MNIC/MCM Device: The TACGWD acts as the logical TCP/IP interface between the host PC, MV Cameras, and all excitation related electronics. (The PoE Switch is the physical layer interface). Machine Vision Cameras (MV Cameras): The Machine Vision (MV) Cameras provide image acquisition and image processing functionality to determine the organism coordinates, size and other parameters related to the organism geometry. Channel Control Modules (CCM): The CCMs provide demultiplexing for excitation related communications between the TACGWD/MNIC and the ECMS. (I.E., each CCM interprets addressed multi-drop network commands for it's corresponding ECM channel). Excitation Control Module (ECM): The ECMs execute excitation-related commands from the CCM and provide control signaling to SCM and ICM hardware accordingly. The SCM and ICM contain no MCUs, so all firmware functionality resides in the ECM. Shock Control Module (SCM): The SCM provides a voltage controlled current source and 6 half-H bridge networks. Together these circuits generate a rotating AC current to drive the 6 electrodes in the Electrode Holder Assembly. Illumination Control Module (ICM): The ICM provides 4 quadrants of blue Negative Reinforcement (NR) Illumination and 4 quadrants of red Back-Ground (BG) illumination. (**B**) The general design includes an illumination head with four quadrants, containing blue and red LED's in each, that illuminates a petri-dish from above. Within each petri dish is a 12-sided electrode holder, hexagonal in design, that can deliver electric shock to the dish based on organism behavior. Below the dish is a red bypass filter (to remove all light except for red, simplifying background subtraction) and an individual camera which records video for each channel. Video feeds and electrical connections from the illumination head and electrode holder run behind the physical device and are processed/controlled by a separate electronics rack, which is in turn connected to a PC for control by the user. (**C**) The physical device is composed of 4 separate banks each containing 3 channels, for a total of 12 testing environments, and is raised off the floor for ease of access. (**D**) A close up of an open bank of channels reveals the illumination head with dividers, electrode holders, petri dishes. The connection running to the electronics rack can be seen in the background. The basic workflow is shown in (**E**). After loading the animals and setting up trial parameters on the master PC, the device runs (in parallel, for each dish independently) a cycle consisting of altering dish conditions (if needed), ascertaining coordinates of centroid of each animal, determining which animals' dish conditions are to be changed based on the trial type (e.g., shock applied as punishment, or lights turned off as reward), and writing current state data to log file. At the end, special scripts process all of the data and produce numerous statistics characterizing the behavior of each channel's subject.

The automated behavior machine consists of a multi-channel experiment environment that is comprised of modules, with each module containing three experimental channels. The system is connected to a single PC running Microsoft Windows™, and the top-level system schematic is shown in Fig. 1A. A single channel is fundamentally a Skinner Chamber, in which one animal receives stimuli, is observed, and is given feedback in the form of light and/or weak electric shock. Our current system has four banks of 3 modules (12 units total), although additional banks can be readily added for higher throughput. The modules are mounted in an aluminum frame, bolted in place, and raised off the ground for ease of access. Each bank of channels contains three Petri-Dish holders (designed to snugly fit standard 6 cm Petri Dishes), three Machine Vision Cameras (Insight-Micro 1400, Cognex Corporation, Natick MA) and three channels of control electronics (Fig. 1B,C).

Each Petri-dish is illuminated by an Illumination Control Module (ICM) that sits 8 cm above the dish, mounted to the top of the Illumination Head, and provides lighting to the Petri-dish in four separate quadrants. The ICM provides blue and red illumination through high-brightness Light Emitting Diodes (LEDs, which allows bright light with minimal heat output, Osram Semiconductors, blue LED; 470 nm, red LED, 635 nm). Between the petri dish and the illumination control module is a “Diffuser/Divider Assembly” attached to the bottom of the Illumination Head. The assembly houses a diffuser plate which ensures even light distribution throughout the dish and supports two stainless steel vanes mounted at right angles to each other to allow independent light status in each of the quadrants of the dish and minimize the leakage of light from neighboring quadrants within the same Petri dish (Fig. 1D). The Petri dish holder insert also contains electrodes (see below). Much attention was given to optical and electrical separation of dishes from each other, to ensure that each animal perceived only the stimuli intended for it. Thus, the dishes have a high degree of isolation with respect to stray light and electromagnetic interference to ensure that the light and shock conditions in one dish do not impact the animals in adjacent dishes. Similarly, high-performance electronics ensure that the timing between behavior and outcome was as small as possible and uniform among all of the dishes (synchronization). A detailed spec of all of the tolerance limits and performance characteristics of the device is given in Figure S1.

### Basic Control Loop

**Figure 2:**
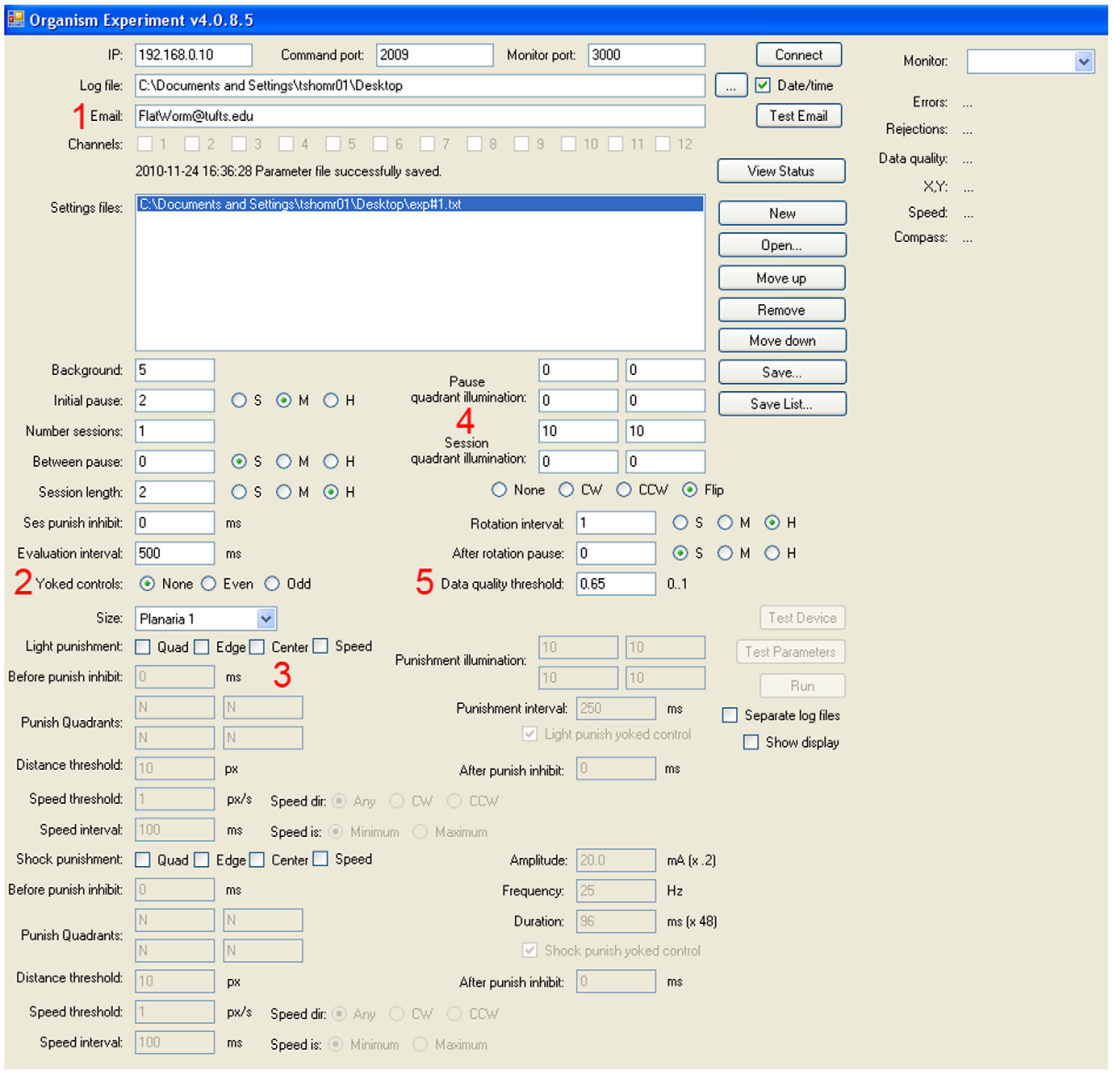
Example of the front end graphical user interface (GUI). Through this interface, the user defines the trial to be performed. Observation-only trials (useful to characterize behavior of mutants or pharmacologically-altered individuals) can be run simply by leaving out any shock or light feedback to the animals. In this example, the GUI is programmed for a planarian phototaxis trial as describe in Fig. 5. Notable features of this GUI include: (1) e-mail notification, both after a successful trial ends and immediately if a serious problem occurs during the trial; (2) yoked control mode can be indicated for half of the device chambers (the 6 even or odd channels); (3) light punishment can be associated with a specific position of the animal -4 quadrants can be set to yes (Y) or no (N) with specific distances from the edge or center or with general movement (speed), and the electric shock conditions can be set similarly (independently); (4) blue light illumination in different intensities can be set as background or punishment for each one of the quadrants; and (5) data quality threshold filter can detect abnormal animal movements like jumps of a large distance in a short time that may indicate a tracking problem. The threshold setting 0–1 will determine when punishment should be stopped in order to prevent false training.

The workflow is shown in Fig. 1E. In a front-end application running on a PC (see Figure 2 for a sample of the graphical user interface), the user describes a set of stimuli and a set of outcomes that will occur if the animal behaves in specific ways. The user interface is flexible, so that almost any conceivable relationship between lights and position of the animal can cause a change in light conditions or a mild electric shock. Thus for example, the user can select for a single lit quadrant out of four, and shock the animal if it does not stay within it; if the lit quadrant moves every so often, the animal is trained continually across days, with no intervention on the part of the experimenter, to follow the moving light. Pauses, yoked controls, and many complex variants of this can be accommodated. Samples of types of trials that can be performed are in Table 1.

Once the parameters of the trial are set and the animals loaded into the machine, the trial begins. Its progress can be monitored on the PC, but it runs unattended, continuously providing stimuli as instructed, observing the position of the animals, changing the light/shock as needed, and recording all of the data in a logfile. An email is sent at the completion of the trial. At this point, the dataset is processed using a custom Excel™ script, which identifies any irregularities (e.g., instances of failed tracking) and produces summary statistics on all of the main characteristics of the experiment. The dataset also contains tracking information as QuickTime movies (so that specific behaviors in any channel can be re-analyzed by hand), as well as static curiosity maps (pseudocolored occupancy plots showing at a glance where each animal spent most of its time during an entire trial).

### Control of Light

**Figure 3:**
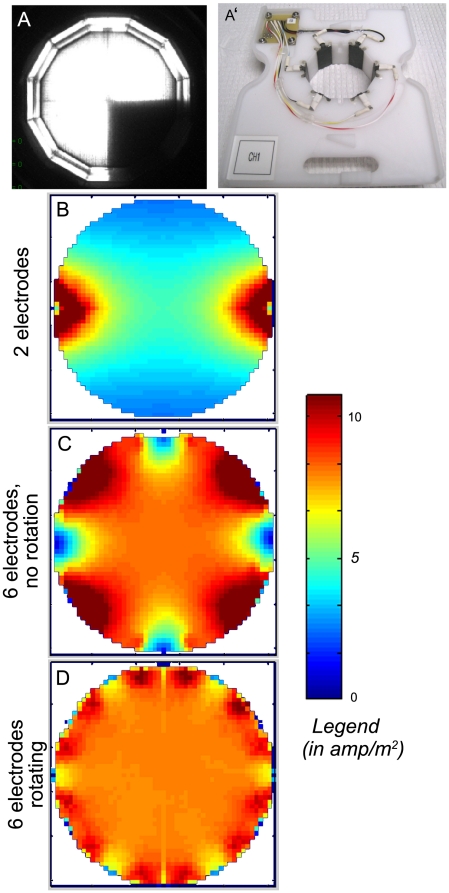
Application of light and electric shock to a Petri dish with aqueous medium. (A) A divider extends vertically from the LED assembly (without touching the water in the dish) and provides for excellent separation between light and dark quadrants (minimal light leakage). (**A'**) The electrode assembly is composed of a round insert composed of white delrin, 6 iridium oxide-coated titanium electrodes, and control electronics. The width and height of the insert were designed for a snug fit with the walls and base of the petri-dishes used in experiments, to ensure planarians could not exit the testing environment from below. When shocks are issued to a given dish holder, two adjacent posts send and receive shock from the two opposite posts at an AC frequency specified by the user. Every 8 ms the source and sink electrodes rotate to the right, with a complete rotation occurring in 48 ms (the shortest shock that can be delivered). To ensure that animals received a nearly identical shock regardless of their position or orientation, finite element analysis was used to model the J field. While there are six physical electrodes, only 4 poles are shown in the model analyses (panels B–D) because only four are active during any given shock pulse. (**B**) A conventional 2-electrode design has a highly anisotropic field density, with hot spots near the electrodes and a defined polarity that will affect animals differently depending on their orientation with respect to the positive and negative poles, and the line connecting them. (**C**) A six-electrode design does better, but exhibits some dead spots as well as hot spots around the edge. (**D**) Much better homogeneity is obtained by using a 6-electrode design in which the electrodes take turns being the positive and negative pole. In this scheme, 82% of the dish area has a current density within 30% of the mean.

Software allows light to be controlled in individual quadrants (Fig. 3A shows the efficiency of the top-mounted divider in establishing distinct light and dark quadrants). Red light is always used as background illumination and is typically set homogonously between all quadrants, while blue lights are turned on as a training or punishment stimulus. A red filter (632 nm narrowband) rests just above each camera effectively removing any blue light from the video feed, even though it is perceived by the animals clearly. This allows the camera to be sensitive to subtle changes in shadow (e.g., a planarian moving along the dish's edge) despite large variations in overall light levels perceived by the animals in different quadrants and dishes. The use of two different colors was driven by the need of the camera to be able to see animals without providing an unpleasant environment for organisms that are negatively phototactic. For example, planaria generally avoid light [103], but have very low sensitivity to light in the red range of the spectrum [104].

Both blue and red lights can vary in intensity from completely off to full brightness in 10 even steps, and the blue lights can be controlled so as to vary as a result of animal behavior (for example, have the blue lights on at a weak intensity when the organism is at the center of the dish, and become brighter when it nears the edge). This allows gradual response in behavior shaping experiments. In addition, the software can cause the lighting conditions to “rotate” 90° or 180° at any interval set by the user. This functionality allows (for example) “chase the light” experiments, where the organism must continually follow a moving quadrant of light to avoid punishment.

### Control of Shock

Electric shock can be applied to individual dishes based on organism position or movement rate over a given period (the latter is designed to facilitate “keep moving” trials). Each plastic insert contains a set of 6 iridium oxide-coated titanium electrodes extending the entire height of the insert (Fig. 3A'). When a shock command is issued by the device, two adjacent electrodes act as a source for the current while the two opposite electrodes act as sinks. Every 8 ms, the sources rotate one electrode to the right, meaning that after 48 ms the source has rotated around the entire dish (this occurs “virtually” via solid-state electronics – no actual movement takes place). The use of multiple electrodes (not simply 2 poles) and the rotation scheme was designed to maximize the homogeneity of the field (Figure S2 contains the models and data needed to perform this analysis on electrode configurations). The electric field produced by a pair of electrodes (used in most such studies in the past) is anisotropic, introducing significant variability in the perceived strength of the aversive stimulus depending on where the animal happens to be. Our scheme minimizes the ‘dead zones’ and ‘hot zones’ (Fig. 3D). Interestingly, some organisms including planaria respond differently depending upon the directionality and polarity of current relative to their own orientation [105]. The rotating scheme ensures that, even within short shock periods, the field is applied across multiple angles ensuring efficient punishment regardless of where the animal is or which way it is facing. The electronics also precisely match the cycles of alternating current flow during rotation, ensuring that it is net balanced at the end of each shock, thus avoiding accumulation of ionic gradients in the dish (e.g., excess of positive or negative charges at any electrode vicinity).

Current can range from 0.2 mA to 20 mA in 0.2 mA increments, and the duration can be any number divisible by 48 ms (the amount of time it takes for the source electrode to rotate completely around the dish). All tadpole training used a 1.4-1.6 mA electric shock, as this was the minimum current which induced a behavioral response. All shocks are delivered as a square waveform with a frequency selected by the user between 10 and 1000 Hz. In addition, punishment can be delayed (for example: when the lights come on, wait 5 seconds and then issue a shock) or inhibited for a given interval (shock under blue quadrants, but after shocking wait 10 seconds before shocking again so the organism has time to move to a different location before the next punishment).

This system offers consistent, balanced shocks to individual dishes based on behavior, with flexible control over duration, intensity, and AC frequency of electric shock. Parameters can easily be adjusted, making the system usable for many aquatic species (current control ensures that shock can be kept constant despite variability in salt concentration of the media). Many different shock paradigms (e.g., continuous weak shock vs. short strong shock) can be tested to determine which one results in the most robust learning for a particular type of experiment. Rounded inserts provide a continuous smooth surface, reducing edges in which organisms can become caught, and easing the cleaning process.

### Software and firmware

The system itself runs an embedded Linux operating system that communicates with the front-end PC through an Ethernet connection. Each of the modules continuously produce message packets containing the X,Y coordinates of each animal (the centroid of its shape) in each dish. These packets are received by a thread within the front-end application, which logs the data, makes decisions based on the position of each animal and the defined trial type, and issues commands to control light and shock as needed. This process proceeds at a rate of up to 25 Hz (25 complete cycles of observe-decide-punish per second), fast enough to train rapidly-moving organisms like *Xenopus*.

Tracking of animal position and speed from the camera images proceeds through 3 stages. The first stage (occurring in firmware on-board the processor at each dish unit) analyzes all pixels in the complete image from the camera, one frame at a time. The second stage performs quality control, rejecting frames that may be erroneous according to several criteria. The third stage calculates the final best estimate of animal position and speed, thus producing the instantaneous coordinate values of the animal in the current frame. It then combines this latest estimate with the history of coordinate values from preceding frames, through double exponential smoothing, to derive the best estimate of position and vector velocity. The second and third stages are performed in the main control code running on the front-end PC.

### Image processing

The camera (Insight-Micro 1400 from Cognex Corporation) contains an internal Digital Signal Processor that executes the image processing algorithms - a sequence of image processing operations to acquire, enhance and analyze the raw image from the camera as follows. The background is removed, leaving the organism as image “blobs” (arbitrary clusters of bright pixels representing an object found from subsequent processing). This system contains an intelligent background update algorithm that automatically captures the backgrounds of empty quadrants as the organism moves about the image. The contrast of the resulting image is adjusted to enhance the brightness level of the organism blobs while generally reducing the brightness of background noise. This system also contains an intelligent contrast adjustment algorithm that uses a “histogram stretch” technique to select an optimal amount of pixels to make bright based on the typical size of the selected organism. A binary threshold is applied to the grayscale result of the contrast adjustment, producing a black & white (binary) image that shows blobs representing the outline of the organisms. The system selects threshold level such that the threshold is higher than the level of background noise. The level is selected based on the statistics of the grayscale image input. A blob selection algorithm is applied to binary result of the threshold operation. This selects only continuous groups of pixels that are above a certain area, and ignores small groups of pixels that are typically noise artifacts. The resulting blobs are sorted and the resulting geometrical parameters (location, size, perimeter, etc) are placed as message packets on the TCP/IP network.

### Filling methods

Standard 60×15 mm petri-dishes (Fisher Scientific) are inserted into each of the channel depressions and secured by the electrode insert, clamped mechanically at opposite ends. Fresh media is added to the individual channels (13 ml for *Xenopus* and *Danio*, 11 ml for planaria), after which each bank of channels is closed and locked; upon completion, background acquisition is initiated by the training software. Following background acquisition, each bank of channels is opened and an individual animal is placed into the center of each dish. When all channels are loaded, the illumination heads are again locked in place and accurate tracking of each animal is inspected by eye using a direct video feed (observed on the monitor of the front-end PC), after which the experiment is executed by the training program.

### Data output

Data for each trial are saved to a single log file in text format, containing positional, lighting, and punishment information for each channel at the specified frame rate. Log files are first processed into individual excel spreadsheets for each channel (animal) in an easy-to-read format, and then loaded into a custom Excel workbook which automates data analysis (e.g, average time in each quadrant by the animal, average speed, proportion of time punished, time spent at the edge vs. center of the dish, and area of the dish explored).

The workbook also calculates “time-segment” data to the user's specification. This time segment function bins the data into any interval specified by the user. For example, it is possible to produce the average movement rate or quadrant location of the organism in five minute, ten minute, or thirty minute blocks to look at changes in behavior over time. These statistics are useful to compare a number of variables including rates of learning, quenching of memory, and exploration/habituation to the testing environment. Time-segment analysis also allows the user to make adjustments based on the organism being tested; the same workbook could be used to evaluate fast moving tadpoles in five-minute increments or slow moving planaria in one-hour blocks. In addition, the time-segment function greatly aids in re-analysis of data. If it is necessary to re-examine particular time periods of a trial in greater detail, the same workbook can be used to generate a new output without the need of creating, copying, and pasting new macros into multiple excel files, greatly reducing the time required for analysis.

### Animal Husbandry

Colonies of planaria, *Dugesia japonica* and *Schmidtea mediterranea*, were stored in 1 L rectangular glass containers, filled with Poland Spring^tm^ natural spring water for *D. japonica* and 1X Montjuïch salts [106] for *S. mediterranea.* Worms were kept on a 10 h/14 h light/dark cycle at 16–17°C. Planarians were fed once per week with organic beef liver and the water was changed three times a week. Planarian were starved a week before use in order to increase activity and to inhibit spontaneous fission. *Xenopus laevis* larvae and *Danio rerio* fry were raised in 100×20 mm petri dishes (Fisher Scientific) at 18° and 24°C respectively, under a 12 h/12 h light/dark cycle and fed daily. Planarian (invertebrate) experiments do not require animal committee approval. All vertebrate experiments were conducted in accordance to accepted NIH and university guidelines and approved protocols (Tufts IACUC protocol number M2008-08).

## Results

### The system development process

We first identified the functional needs of this system by careful analysis of typical projects in the field, as well as the biggest roadblocks keeping behavioral analysis out of reach of typical molecular biology/development laboratories. We examined commercially-available systems and determined their limitations and capabilities. We worked closely with a local engineering firm, Wireless Techniques, to develop the necessary software, hardware, optics, electronics, and programming components. It was crucial to use one single entity for the construction of the device; although the individual subsystems can be outsourced, the necessary tight integration cannot be achieved unless the whole system is designed to work as a coherent platform from the outset.

During the development phase, a number of problems were solved, including the following:

1. A high sensitivity current detector was designed to operate in the presence of high common-mode voltages for shock sensing; this is key, since a feedback needs to tell the device that a given shock actually occurred (some organisms secrete slime or change the salt content of the chamber's medium, which in turn impacts the amperage of the shock current).
2. The lighting system required extensive illumination head sealing for 72 hrs continuous run time, and a high-performance LED cooling system for 50,000 hour life requirement.
3. A system of signal switching was implemented, to allow shock to be administered in a shifting hexapole configuration, producing a uniform electric field in the dish and insuring the animal receives an appropriate shock regardless of orientation relative to the electrodes. This required finite element analysis modeling to design a chamber geometry, electrode position, and voltage profile which ensured a homogenous-current distribution during punishment that gives no dead-spots in which to hide, nor hotspots that will be avoided independently of the task being learned. All electronics were designed so as to minimize vibration and noise that could be confounding factors for sensitive aquatic animals.
4. Electrode materials were chosen in a shape, size, and material that balances nonlinear tradeoffs of low capacitance, lack of hot-spots (non-uniformity of field), and avoidance of electrolysis products (toxic metal ions) being shed into the medium during shock. The electrode holder was also chosen for inertness (low toxicity) and optimal compatibility with the electrodes, including also a spring-pin connection system for easy disassembly.
5. Image processing algorithms were designed and optimized to allow organisms to be tracked reliably despite significant variation in size and position relative to the walls and divider. This is a very challenging task because of the many shadows, edges, and occasional bubbles, the ability of animals to move within the Z plane (vertically), and the huge range of brightness levels (gain) between quadrants in which no blue light is on and those in which it is on at full strength. Moreover, the software had to continue tracking even when water levels changed over 3-day experiments, which is difficult because as the meniscus moves due to microevaporation, additional shadows and shifting brightness levels impact the use of a background image captured without the animal at the start of the experiment. An automatically-healing background capture algorithm was developed to derive background images from any quadrant vacated by an animal, as well as an intelligent image-processing component that adjusts parameters as a function of organism size. Figures S3, S4 and Videos S1, S2 contain examples of observing real animals, showing successful tracking under normal and difficult conditions.
6. Software was written, containing a flexible Graphical User Interface (to allow the operator to define the type of trial and controls he or she wishes to perform, Fig. 2) and front-end code, and firmware to track the animals (image processing and machine vision tasks) and control the light- and shock-emitting electronics to the chambers. This allowed individual shock and light levels (each animal needs light and shock inputs specific to its own position and performance in the past time intervals, for true flexibility of training paradigms). This included the development of an IR light source and cameras, a staggered processing and synchronization scheme, registration of images to a small tolerance to allow background subtraction, high-gain image analysis (to allow detection of animals in both dark quadrants and lit-up quadrants), diffraction and light leakage due to quadrant separators, implementation in firmware on digital signal processor chips, and much more. Video S2 contains an example of an animal's tracks re-created after the experiment (from saved logfile data) using one of the utilities developed for this system.

The work was performed in 3 phases. First, a single-channel prototype was created and tested to ensure that environmental controls and tracking were optimized. Next, a 3-channel system was multiplexed to test parallelized independent function and lack of “leakage” of stimuli between adjacent cells. Finally, the whole 12-channel system was assembled and fully tested for compliance with the original specification document. Below, we illustrate the device's use through proof-of-principle analysis of behavior in 2 different organisms.

### Location preference in tadpoles

**Figure 4:**
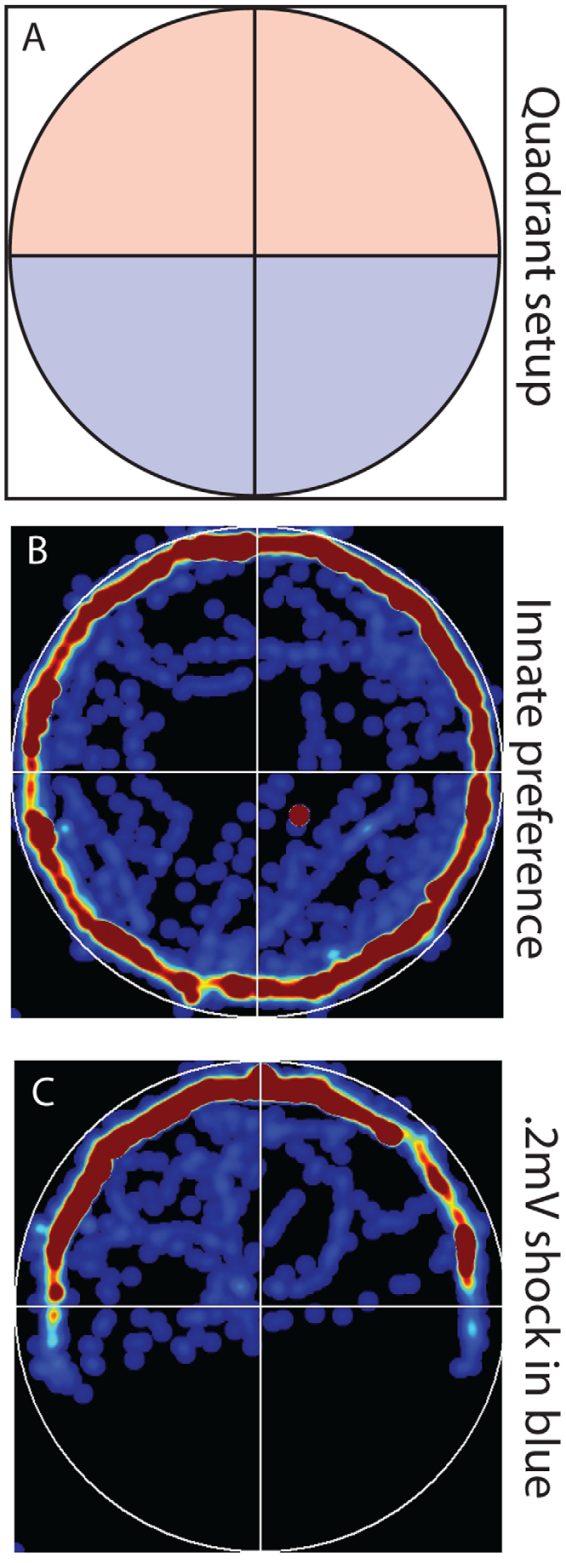
Occupancy maps generated from *Xenopus* tadpole behavioral data to evaluate overall positional trends during trials. (**A**) Different quadrants of the dish can be set with varying light conditions (color and brightness), for instance half of the dish in red light and the other half in blue light. (**B**) Curiosity plots demonstrate overall preference for both light color and dish location (such as the edge or center of the dish), as illustrated by trials showing that tadpoles have no preference for either red or blue light. (**C**) When tadpoles receive a 1.4 mA electric shock under blue light, individuals now spend almost all of their time under the red illuminated half of the dish. Higher intensity of color (red) in heat plot indicates more time spent in that location.

A goal of all automated behavior analysis systems is to be able to rapidly screen basic behaviors of organisms, including preferences for locations, lighting conditions, and general activity levels. A useful feature for analysis of trial data is the “curiosity plot,” a pseudocolored heatmap showing the relative frequency of occupancy for each position in the dish. To illustrate, a 14-day-old *Xenopus* tadpole was placed in the device with the top half of the dish illuminated with red light and the bottom half of the dish illuminated with blue light (Fig. 4A). The curiosity plot software reads in the position of the tadpole over the 30 minute trial and places a blue spot (size determined by user) on a map of the dish for each timepoint. Where dots overlap (indicating multiple visits to this location by the animal during the trial), the color increases in intensity, from blue to green, yellow, then red. The output shows that over the course of a 30 minute trial the tadpole spends the majority of the time at the edges of the dish with no obvious preference or blue or red illuminated halves (Fig. 4B). To screen for effective levels of shock (those that illicit a behavioral response but do not injure or kill the organism over long periods of exposure) in different organisms, heat plots can be used to determine if tadpoles avoid quadrants that are punished. Tadpoles receiving a 1.4 mA shock when located in the blue half of the dish quickly learn to spend the majority of their time on the non-punished red side (Fig. 4C). Heat plots can be generated within minutes of trial completion and provide a convenient way to evaluate light preference as well as responses to varying intensities and durations of shock.

### Planaria training

Planarian learning and memory, and the mechanisms underlying it, have been studied since the beginning of the 20^th^ century, mainly through the 50's and 60's by McConnell and his associates [105], [107], [108]. Although the extensive research led to some remarkable discoveries [105], [107], the field of planaria learning and memory has been limited in impact, suffering from controversies mainly due to the lack of sufficient standardization of experimental procedures [108], [109]. Our platform was designed to overcome these problems, which will resolve the controversy through quantitative rigorous behavioral analyses and standardization of experimental procedures (greatly facilitating the reproduction of experimental results by other labs).

Here we describe preliminary studies characterizing suitable planaria species for automated training. We examined two planaria species, *D. japonica* and *S. mediterranea*. Both species are easy to breed and maintain in the lab, possess outstanding regenerative capabilities and have been well studied in recent years using modern cell and molecular biology techniques [45], [110], [111]. In contrast to *D. japonica*, *S. mediterranea* has a stable diploid genome making it more amenable to genetic approaches [112].

In each of the experiment trials, we utilized the device to track locomotor and exploration behavior, as well as light preference, of 12 planarians simultaneously. A total of 24 worms from each species (0.5–1 cm in length) were tested in 4 separate trials (results are displayed as the average of the entire group for each species). Each trial lasted 122 minutes. During the initial 2 minutes, the environment was illuminated with a homogenous red light background. Following this initial period, one half of the training environment was illuminated with blue light and the other half remained red. After one hour the illumination halves were swapped.

**Figure 5:**
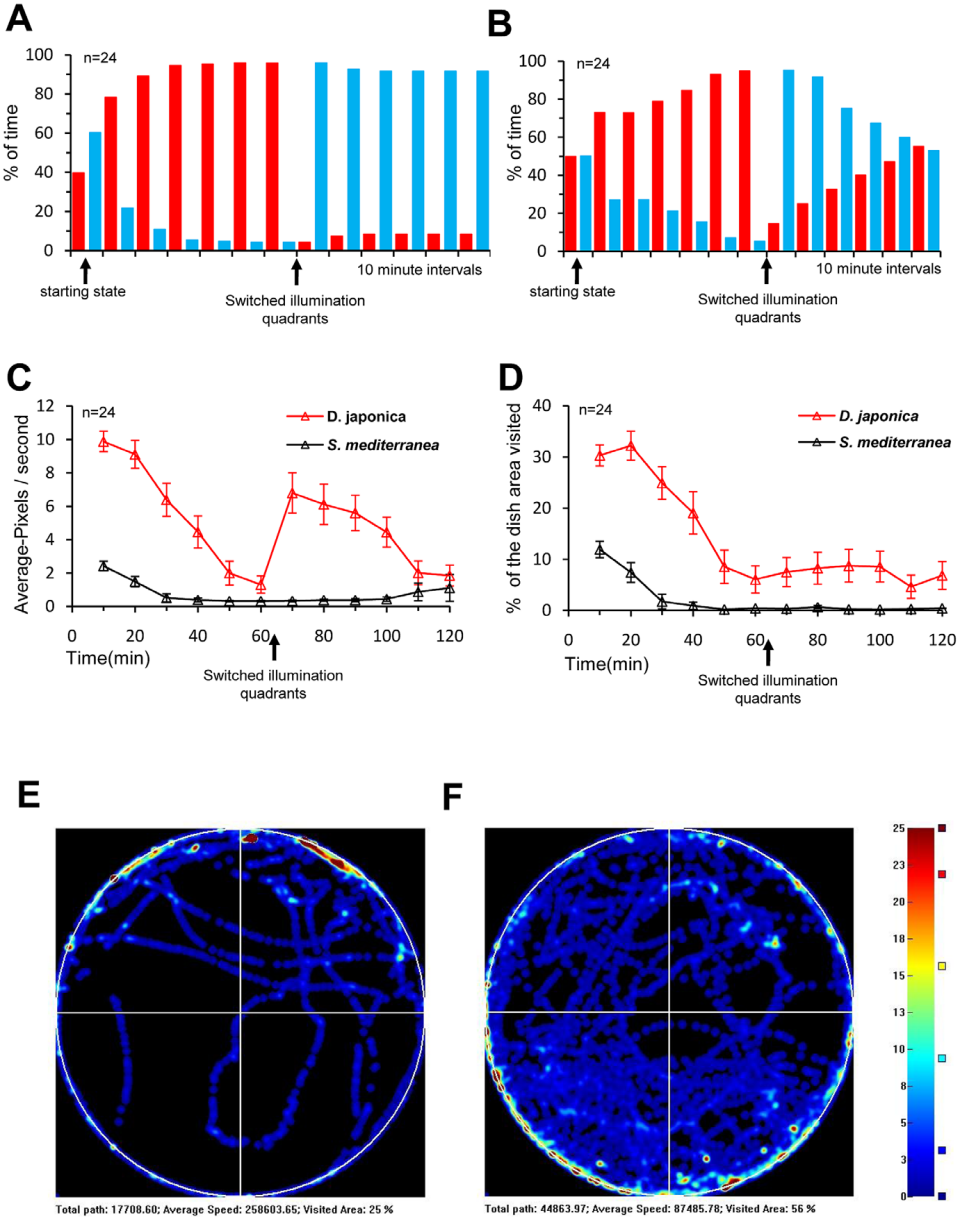
Comparison of planarian exploration behavior and light preference between *D. japonica* and *S. mediterranea*. During the trial one half of the training environment was illuminated with blue light and the other half with red. After one hour, illumination quadrants were exchanged so that the red half was now blue and vice versa (see arrow A–D). (**A,B**) Preference to light, the histograms summarize the percentage of time spent by the overall worms from each species (*S. mediterranea* in **A** and *D. japonica* in **B**) stayed in the red or blue illuminated half. Each category bar represents an average of 10 minutes, except for the first category (see arrow) that indicates the initial position of the worms just before the start of the trial, when all dish quadrants were illuminated by red light. Both species preferred the red light and were located in this half by the end of the exploratory phase (n = 23/24 *D. japonica*, n = 23/24 and *S. mediterranea* respectively); afterwards, they quit moving and settled down. Reversing the color light in each half induced just some of the subjects (13/24 of *D. japonica* but only 2/24 of *S. mediterranea*) to move into the red illuminated half. (**C**) Comparing movement rates and (**D**) explored area, both species demonstrated an exploratory phase, after which movement rates and exploration dropped to low levels. *D. japonica* (red triangles) showed significantly greater average speed and area explored than did *S. mediterranea* (black triangles). (**E** for *S. mediterranea* **, F** for *D. japonica*) Occupancy maps enable the ready evaluation of overall positional trends during trials. The plots generated from the behavioral data of the most active worm from each species during the first hour of the trial are shown. The red illuminated half is up in E and down in F, both indicating a preference for red over blue light as well as for the edge of the dish.

The results show that both species prefer red background illumination over blue (Fig. 5). At the end of the first two minutes of the trial (where the whole environment was illuminated with red light), the worms were found scattered uniformly (Fig. 5A,B, starting state). Shortly after half of the dish was illuminated with the blue light, worms started moving from the blue light to the half illuminated with red light; at the end of the first hour most worms (23 out of 24) from each group were located in the red half. Examination of the last 10 minutes of the first hour reveal that both species spent approximately 95% of their time in the red illuminated half (Fig. 5,A,B). After the illumination quadrants were exchanged, the *D. japonica* worms slowly moved to the red half, however the majority of the *S. mediterranea worms* stayed in their original locale— previously illuminated with red but now illuminated with blue (Fig. 5.A,B). At the end of the second hour, 13/24 *D. japonica* but only 2/24 *S. mediterranea* were found in the red illuminated half (as a group, *D. japonica* spent 55% of the time in the red half while *S. mediterranea* spent only 8.17% during the last 10 minutes of the trial, Fig. 5 A,B).

These light preferences were further examined by looking at exploration behavior (Fig. 5.C–F). The data on movement rate and total area explored during the trial showed that both species exhibited an exploratory period during the early phase of the trial when they were first introduced to the new environment (Fig. 5C,D). However, *S. mediterranea* displayed a significantly lower movement rate and shorter exploratory phase (10–30 minutes) compared to *D. japonica* (20–50 minutes). At the end of this exploratory phase, the worms settled down in their preferred place (the red illuminated half, usually on the edges of the dish). When the blue and red illuminated halves were exchanged after the end of this exploratory period, it caused the *D. japonica worms* to move into the new red half, while most of the *S. mediterranea animals* stayed where they were (despite now being in the blue half). The curiosity maps (Fig. 5E&F) of the one most active worms from each species show that *D. japonica* is much more active and its exploration behavior is more extensive. It also reveals that both species spend most of their time at the edges of the dish (red spots in Fig. 5 E&F).

These data show that the system is capable of accurately tracking animals as small as planarians 0.5 cm in length. As has been observed in manual studies, planaria indeed show negative phototaxis [103], although they have a lower sensitivity to red light [104]. Thus, combinations of faint red background and bright blue light in our device can be used for planarian training paradigms such as training (against their normal preference) to move toward the quadrants illuminated with blue light or instead illuminating the entire environment with blue and using its removal as a reward for correct behavior. *S. mediterranea* locomotor and exploration behavior are markedly less pronounced than those of *D. japonica* and as such *D. japonica* is likely to be a better candidate for learning studies. Using *D. japonica,* which can readily be manipulated even after the initial exploratory phase, will allow long (up to 72 hours) trials and flexible training procedures uniquely suited to this automated system.

### Comparisons of behavior in 2 vertebrate model organisms: tadpoles and zebrafish fry

Applicability of the system to a range of aquatic organisms enables comparisons between related or divergent species, as well as the same species at different ages. As an example, we compared the preference for blue or red light and movement rates of 14-day-old *Xenopus* tadpoles and 21-day-old juvenile zebrafish. These ages were chosen based on the relative size similarities between the organisms at this stage. For the comparison, both organisms were put in a testing environment where half of the dish was illuminated with red light, while the other half was illuminated with blue light. Over the course of 30 minutes, quadrant location and movement rate were recorded at 10 frames per second (10 Hz) for each organism and averaged over 5 minute intervals.

**Figure 6:**
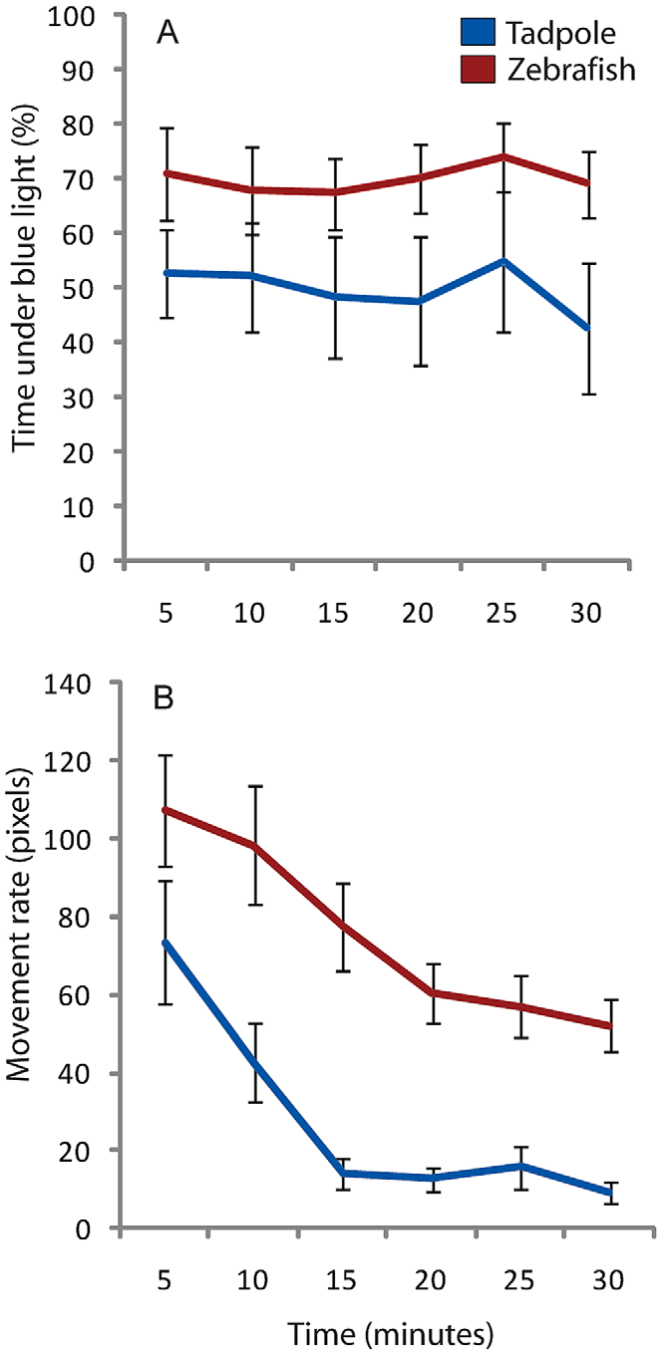
Comparison of color preference and movement rates between 14-day-old *Xenopus laevis* tadpoles and 28-day-old *Danio rerio* fry. Tadpoles and fry were placed in the device with one half of each testing environment illuminated with red light and the other half illuminated with blue light. Images were recorded at 10 frames per second to accurately measure the movement rates of each organism. (**A**) Over a 30 minute initial preference trial, *Xenopus* tadpoles showed no preference for either blue or red light, while *Danio* fry spent more of their time under blue light. (**B**) Comparing movement rates, both organisms demonstrated a 15–20 minute exploratory phase, after which movement rates became stable, with zebrafish showing a greater average speed than *Xenopus*. N = 9, 12 for *Danio* and *Xenopus* respectively, error bars indicate ±1 SEM.

The data reveal that while *Xenopus* tadpoles showed no preference for either color light, spending roughly half of the time in both blue and red halves, zebrafish spent approximately 70% of the time under the blue light (Fig. 6A, t test p = 0.032). In addition, zebrafish also appeared more active, moving around the dish at greater speeds than the tadpoles during the course of the 30 minute evaluation (Fig. 6B). Interestingly both organisms showed a 15–20 minute exploratory phase during the beginning of the trial, with movement rates starting high while the tadpoles and fry adjusted to the training environment. After this initial period, movement rates remained steady (up to 2 hours of evaluation, data not shown).

These data demonstrate the system is effective for comparing the behavior of different organisms of varying shapes (for example, to compare nootropic compounds in multiple species to show conservation of effect across clades and rule out or identify species-specific effects).

### Color conditioning with shock in *Xenopus* tadpoles

While zebrafish have been shown to be good learners in classical conditioning experiments, learning and memory in *Xenopus* has been far less studied. Our learning trial was broken into three separate blocks: an initial preference phase, a training phase, and a testing phase. During initial preference evaluation, half of the dish was illuminated with low intensity red light and half with high intensity blue light. During training optimization experiments, we found that pairing wavelength and intensity as training stimuli resulted in more robust learning than using either individually. Every ten minutes, the pattern of light rotated 90° in a clockwise direction, for a total of 3 rotations over the course of the evaluation (with the lighting conditions at the end of the trial being the inverse of those at the beginning). Location of the tadpole was recorded 10 times a second (10 Hz capture rate) and data was averaged over 5 minutes for ease of analysis and presentation.

The training phase proceeded exactly as did the initial preference evaluation, with the exception of a 1.2 mA AC shock being delivered if the tadpole was in the low intensity red half of the dish, thus conditioning the organism to stay under the blue light. The light was rotated 90° clockwise every 10 minutes during the trial to prevent tadpoles remaining motionless in the red half of the dish without actually experiencing an electric shock, and thus only appearing to learn. During this phase, four identical training sessions of 30 minutes were executed, separated by 90 minute rest periods where the entire dish was illuminated with blue light. Following the final 90 minute rest session, tadpoles were tested for light preference with the exact same setup as the initial phase, where occupancy in neither the red nor in the blue half of the dish was punished.

**Figure 7:**
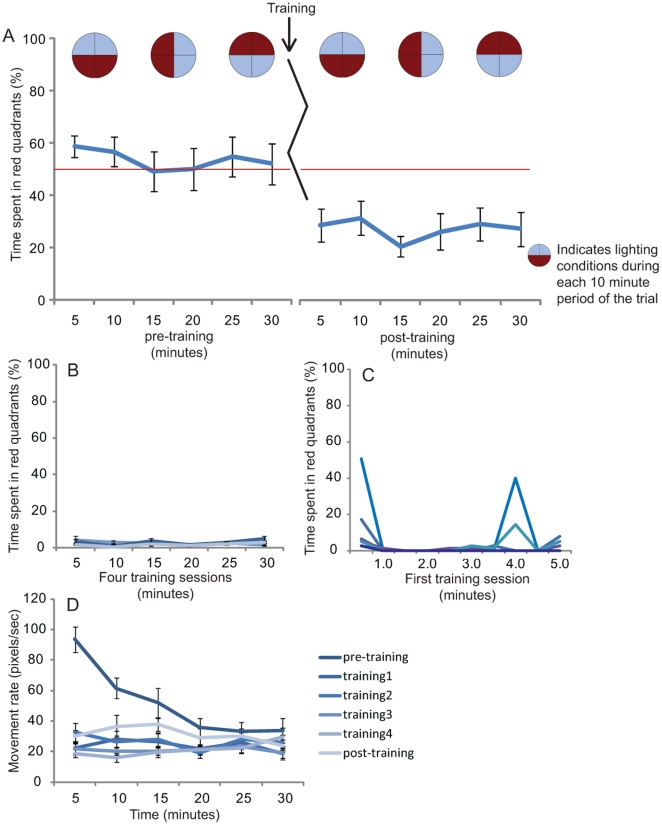
Simple learning trial with *Xenopus* tadpoles. (**A**) Tadpoles 14 days of age were placed into the device individually, with half of the dish illuminated with low intensity red light and the opposite half illuminated with high intensity blue light. The location of each tadpole was recorded and pooled over five min time intervals across a 30 min evaluation. To ensure that increased occupancy of the target quadrant could not be due to simple inactivity, every 10 minutes, the light pattern rotated 90° in a clockwise direction (see diagrams, top of graph). Following initial preference evaluation, tadpoles received red aversion training by punishing individuals in red quadrants with a 1.2 mA electric shock. Conditions were identical to the initial preference phase; lights were rotated 90° every 10 minutes during a 30 minute session. Tadpoles were subjected to four identical training sessions followed by 90-minute rest periods under blue light. Following training, tadpoles were reassessed for light preference exactly as in the initial phase, with no quadrants being punished, and showed a significant difference from untrained behavior, spending more time under blue quadrants (2way repeated measure ANOVA P<0.001). N = 24, error bars indicate ±1 SEM. Red line represents no preference for low intensity red light or high intensity blue light. (**B**) During each of the 4 training phases, tadpoles strongly avoid the punishing red half of the dish. (**C**) Examination of the first five minutes of the initial training session reveals that tadpoles move to non-punishing quadrants within the first 30 seconds (6 individual tadpoles shown). (**D**) Movement rates show an increased ‘exploratory’ phase during the first 30 minutes of the trial (initial preference phase) but remain relatively constant across training and post-training sessions. N = 24 for A, B, D and 6 individuals for C, error bars indicate ±1 SEM. Red line in (A) represents no preference for low intensity red light or high intensity blue light.

Results showed that similar to the *Xenopus*/zebrafish comparison, tadpoles initially possess no preference for either low intensity red or high intensity blue light at the beginning of the trial (Fig. 7, pre-training). However, following four training sessions in which the red quadrants were punished, tadpoles showed a significant change in behavior, spending more time in the high intensity blue half of the dish (Fig. 7, post training, 2way repeated measure ANOVA P<0.001) of their time in the high intensity blue half of the dish. This behavior was not due to individuals remaining motionless during the trial as overall blue preference was maintained even after light rotation at 10 and 20 minutes respectively.

Contrary to our initial expectation of a characteristic ‘learning curve’ across time, we observed that tadpoles appear to avoid shock very quickly and efficiently. Examination of each of the 30 minute training sessions revealed that tadpoles spend near 100% of the time in the blue half of the dish (Fig. 7B). During first 5 minutes of the initial training session (analyzed in 30 second intervals), all individuals moved to non-punishing quadrants within the first 30 seconds (Fig. 7C, six representative individuals plotted for clarity), and this was reflected across all training periods.

Movement rates did not change as a result of training. During the pre-training period, individuals showed the previously documented “exploratory phase”, with a gradual decrease in movement rate as the trial proceeds (Fig. 7D, compare with 6D). After leveling out, it remained steady across training and post-training phases (Fig. 7D). For comparison, we also performed a sham training trial which proceeded exactly as above but in which no shocks were delivered. Movement rates between the sham and training trial were not significantly different (data not shown) suggesting our punishment regime *per se* did not significantly affect movement rates in *Xenopus* tadpoles.

These data, and the fact that no learning is observed when lights are not rotated, suggest the following description of tadpole learning dynamics in this kind of trial. When tadpoles are punished, they move until punishment ceases, then remain in one area while moving slowly until they receive another punishment. Given the speed at which tadpoles can move (they can circle the dish in 2–3 seconds), this occurs extremely rapidly. In the case of non-rotating light trials, individuals then remain relatively still throughout the entire training session and thus receive few punishing ‘experiences’. However, when the lights are rotated during a training trial, it forces tadpoles to occasionally occupy punishing quadrants throughout the trial, which increases the number of incidents in which individuals are presented with the shock-red light pairing, resulting in more robust learning results. Thus, in non-rotating-light trials, tadpoles are learning a “don't-move” behavior, and in post-training evaluations could simply stay in one quadrant even in the event of changing light conditions. In contrast, an association is clearly made in the training outlined above using rotating light: if tadpoles were simply learning ‘don't move’, the preference line would not stay significantly below 50% while light conditions reversed continuously.

These results are promising considering the minimal training period used in this example. It is likely that repeated training regimes over the multiple days would yield enhanced learning, as would pre-screening animals for ‘strong’ or ‘weak’ learners. Future efforts should test training regimes involving a variety of parameters such as position at the center or edge of the dish, moving at a greater or slower speed than baseline rates, or training to intensity of light rather than wavelength. Training to multiple parameters will likely give insight into the functional constraints of tadpole memory. Can a tadpole learn to not move under red light and avoid the edge under blue light? How quickly can preferences for red or blue light be acquired and reversed through training? Will tadpoles with artificially-expanded forebrains, duplicated CNS structures, increased proportion of serotonergic neurons, or ectopic eyes exhibit faster learning, better sensory acuity, or different baseline behavior than wild-type siblings? It is likely that such memory assays can be used as a powerful screening tool for nootropic compounds (cognitive enhancers). All of these questions can be addressed in a quantitative, non-biased manner using the automated training paradigms described above.

## Discussion

The current challenge of modern cognitive science is to understand the processes that span from developmental genetics to the information processing mechanisms that give rise to behavior and thought. The biomedical aspect of this program includes the search for useful neuromodulatory drugs, as well as the restoration of normal cognition as part of regenerative medicine targeting injuries of the CNS. Fundamental advances thus require the characterization of behavior in a variety of genetically and pharmacologically-modified organisms. Manual analyses of animal behavior places significant limitations on experimental progress. These restrictions include the limited number of animals that can realistically be analyzed by hand, the confounding experimenter effects inherent in manual handling and observation by different individuals, and the difficulty of documenting raw results completely enough to enable other groups to analyze all of the primary data and potentially uncover trends missed (or not even recorded) by the experimenters.

Indeed, these problems have been central to a number of controversies in neurobiology. For example, the lack of consensus on the learning ability of planarian flatworms was due in large part to the small sample sizes necessitated by the tedium of training worms by hand, as well as the inevitable but often important small differences in handling by different experimenters (observer bias, oversensitization from handling), inconsistencies in protocols and controls, and difficulties in making every aspect of the data available to other groups in the field [24], [61], [105], [113], [114], [115], [116], [117], [118], [119], [120], [121], [122], [123]. The use of an automated system would have allowed other labs to reproduce even complex behavioral experiments precisely, and analyze the data without bias.

Molecularly-tractable model organisms promise the greatest insight into cognitive function. *Xenopus laevis* larvae have been a popular behavioral system for the investigation of responses to light and gravity, and in individual behaviors and schooling [62], [124], [125], [126], [127], [128], [129]. Because of optical, developmental, and genetic accessibility of neurons and their embryonic precursor cells, zebrafish are an excellent system for investigating how neural circuits give rise to behavior [130], [131], [132]. They have been used to study circadian rhythms [133], prey tracking [134], social interactions [135], and vision [136]. Comparisons of behavior in wild-type and genetic mutant zebrafish have been initiated [130], [131], [134], [135], [136], [137], [138], [139], [140], as have analyses of drug effects on fish behavior, including ethanol [32], [130], [141], [142], [143]. One of the most exciting next frontiers is the synthetic modeling of how neural function gives rise to behavior [144], [145], [146], [147], [148], [149], and an automated paradigm for analysis will greatly speed up this effort. This is of particular relevance to non-rodent model organisms popular in neurobiological studies [150], [151], [152].

We describe the prototype of a modular, highly-flexible, second-generation system that allows quantitative characterization (behavioral and sensory phenotyping) as well as individual feedback (memory and learning studies) using several different modalities. It can be extended to work with almost any other animal type within the relevant size range. Our goal is to ensure that the prototype system becomes widely available and accessible to any lab wishing to perform quantitative behavioral analysis. We are working with engineering firms to (1) modify the design to achieve a polished, user-friendly, relatively bullet-proof, optimized version of this system that can be deployed readily outside our lab as a standard piece of equipment (including for use with students in educational institutions), and (2) set up a distribution channel within which several versions of this system, operating manuals, construction kits, and technical support can be obtained.

It is important to note that while the price for end-users (now that many engineering problems have been solved) will be reasonable (approximately the same as a laser microscopy system), the capabilities of our prototype system were constrained almost entirely by initial R&D costs. Thus, we reached no fundamental limits, and increasing performance along almost every dimension of this system can be readily achieved with further development. Extensions of this system may include: 1) placement of transparent mazes within dishes, and various haptic coatings on the dish surfaces, 2) use of smaller model systems (*C. elegans*), or tracing of specific behaviors (subtle tail twitches or specific kinds of motions) with higher-resolution cameras, 3) tracking animals by fluorescent tags and control of optogenetic reagents [153], [154], [155], 4) addition of microvibration as a stimulus modality, and 5) microfluidic ports for training to odors or exchanges of pharmacological compounds during experiments. The system is readily scalable, and core facilities would be able to obtain large units for high-throughput studies. A significant addition to be developed for this application would be automated animal loading. Solutions for automated dispersing of small organisms exist already, such as the COPAS system [156], and the next generation of screening robots will be integrated with such delivery platforms to enable large numbers of animals to be efficiently placed into the device without human intervention.

Current efforts in the discovery of novel genes, proteins, or chemical reagents that have interesting, useful, and/or enlightening effects upon living systems are focused on screening approaches: locating valuable reagents by large-scale, parallel, automated examination of candidate molecules present in a combinatorial "library" [157], [158], [159], [160], [161], [162], [163]. A number of academic and commercial pharmaceutical projects have generated genetic, proteomic, or small-molecule (drug) libraries that must be screened to identify compounds of interest to both biomedicine and basic biology. Examples include searches for drugs that inhibit particular enzyme pathways in human disease [164] or proteins involved in specific patterning events in developing embryos [165], [166], [167], [168], [169], [170], [171], [172].

There is an enormous list of potential targets for which screening of libraries would result in medically-valuable reagents; similarly, many biological processes, when perturbed and characterized, can readily be used as assays that might lead to a better understanding of endogenous control mechanisms. The crucial and usually most difficult aspect is the choice of screening method. This requires a tractable yet relevant model system and a degree of automation (to ensure temporal and financial feasibility). Some screens—such as those for tumor suppressant drugs—have been successfully conducted using cell culture assays or unicellular organisms such as bacteria or yeast [173]. However, many targets of interest are relevant only in the context of complex organisms (such as the search for memory-enhancing drugs or gene products which participate in specific behaviors, for example). Large-scale behavioral screens in otherwise popular model systems such as mice are not feasible due to cost constraints. Model organisms such as *Xenopus* and zebrafish are ideal because they offer complex vertebrate systems with high biomedical relevance as well as being readily amenable to state-of-the-art molecular, cell, and neuro-biology techniques. Screens on multicellular models such as zebrafish [174], [175], [176], [177], [178], [179], [180] have been successful in cell-biological assays, but the lack of a parallelized, automated system for analyzing memory and individual responses to stimuli precludes effectiveness in high-throughput neurobehavioral screens. The worm *C. elegans* has been utilized for high-throughput automated screens, but this has been in a cell-biological context (measuring death vs. survival), rather than in a behavioral one [181]. One nice counterexample, which illustrates the potential of this approach but is not available in a form generally applicable to other kinds of experiments, was used in a high-throughput fish screen for the perception of auditory stimuli [182]. It is the lack of a scalable, powerful, well-characterized system for parallel investigation of memory and learning in vertebrate organisms that has hampered the commercial screening for new classes of cognitive enhancers or “nootropics” [183].

Automated systems will enable cognitive scientists to ask questions that are currently difficult to address, and will significantly lower the barrier for developmental neuroscience labs embarking on projects that require them to quantitatively characterize the neurobehavioral consequences of alterations in embryonic development pathways. Likewise, pharmaceutical efforts using small animal models to identify compounds or genes with specific neurobiological effects [184], [185], [186], [187] would be able to use scaled-up versions of such automated platforms to perform high-throughput screens for complex behavioral or neurological outcomes. Optical, microfabrication, and computer technology are progressing with increased rapidity and will further potentiate the capabilities of such machine vision and environmental control systems. The advances in basic science and neuromedicine that will result from the widespread availability and increased sophistication of automated behavioral screening are truly exciting to contemplate.

## Supporting Information

Figure S1 Detailed specification of all parameters of the device and its functionality.
(0.03 MB XLS)

Figure S2 Compressed.ZIP archive of the code necessary to perform shock homogeneity analysis on electrode configurations. The analysis objective is to determine the j-field (current density and its uniformity) of electric shock in water for a given electrode and shock configuration. This is done by the software QuickField 5.7 and a custom Matlab script. The assumptions were: a simple (resistive) impedance model, a homogeneous vertical field is assumed (since the electrodes extend down the entire vertical length of the petri dish), and a fixed voltage (+/- 20V) excitation applied across the electrodes.
(0.10 MB ZIP)

Figure S3 Sample images of tadpoles within the device. (A) Tadpoles are easily tracked when in the middle of a quadrant. (B,C) The image processing algorithms are able to detect tadpoles even when located parallel to the edge of the dish, where they are hard to find by eye, due to the sophisticated background subtraction and healing techniques. The red circle with cross-hairs indicates centroid of the animal shape.
(0.21 MB PDF)

Figure S4 Sample images of planaria within the device. (A) Planaria are easily tracked when in the middle of a quadrant. (B,C) The image processing algorithms are able to detect planaria even when located parallel to the edge of the dish, where they are hard to find by eye, due to the sophisticated background subtraction and healing techniques. The red circle with cross-hairs indicates centroid of the animal shape.
(1.21 MB PDF)

Video S1 Sample movie showing real-time tracking of 3 channels' animals.
(5.93 MB MOV)

Video S2 Sample movie showing processing of saved tracking data to recreate the trajectories.
(1.54 MB AVI)
