# Correction: A Second-Generation Device for Automated Training and Quantitative Behavior Analyses of Molecularly-Tractable Model Organisms

**DOI:** 10.1371/annotation/f5985f4c-c70a-498d-887f-9c9635c0ef77

**Published:** 2011-01-07

**Authors:** Douglas Blackiston, Tal Shomrat, Cindy L. Nicolas, Christopher Granata, Michael Levin

Extraneous text was included in the Competing Interests section during the copyediting process. This section was meant simply to state that there was no commercial sponsorship of the reported work and there are no competing interests. The correct Competing Interests section should read: "The authors hereby confirm that the company affiliation of Christopher Granata does not in any way alter their adherence of all the PLoS One policies on sharing data and materials. This company was not a funder or sponsor of the research and does not affect the project in any way. Christopher Granata was paid for his participation in the project off a grant, as were the post-doc fellows involved, and is simply an engineer without academic affiliation. No commercial sponsorship/funding/involvement was involved in this study."

