# Supplementary figures and images for: A Second-Generation Device for Automated Training and Quantitative Behavior Analyses of Molecularly-Tractable Model Organisms
Source: PLoS One. 2010 Dec 17;5(12):e14370. doi: 10.1371/journal.pone.0014370 (PMC3003703; doi:10.1371/journal.pone.0014370)

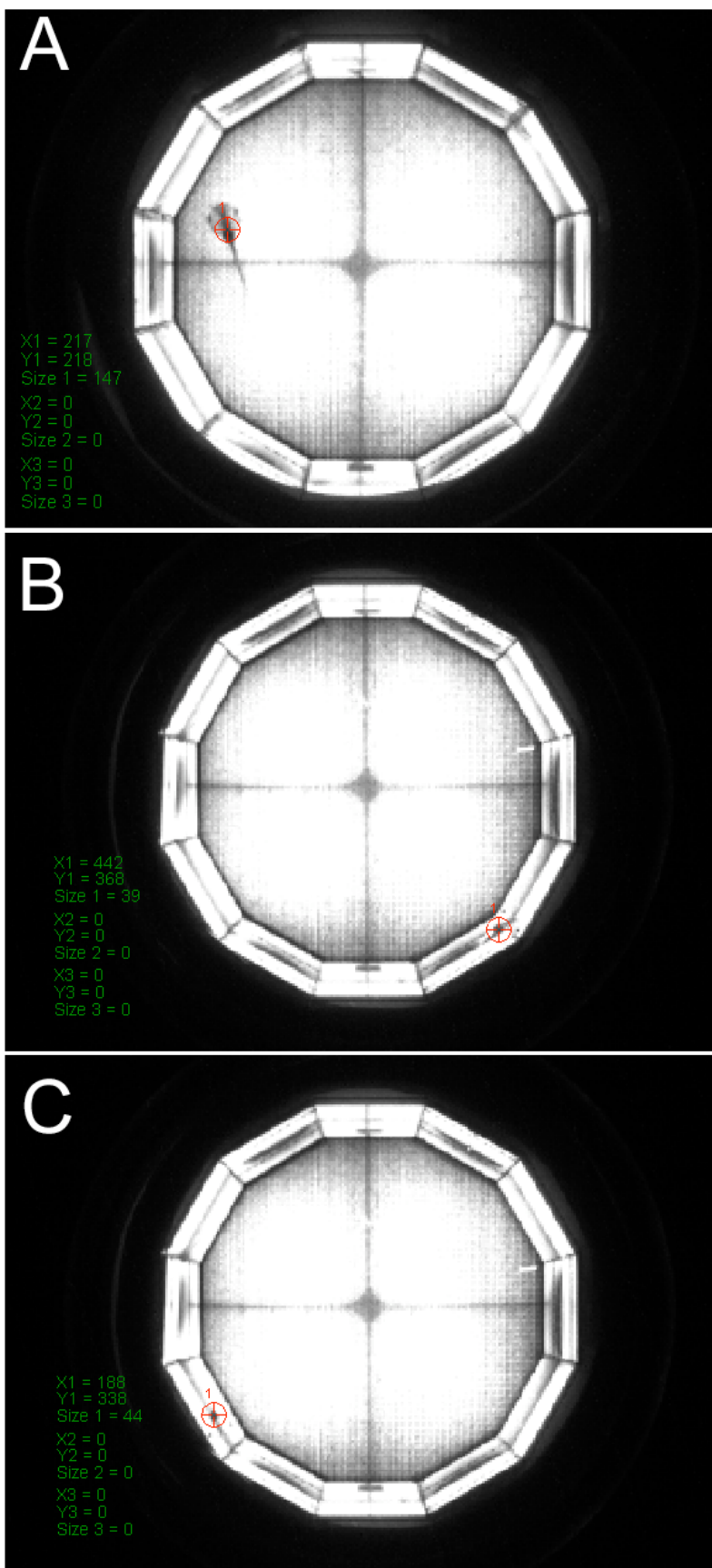

Supplement: Figure S3 — Sample images of tadpoles within the device. (A) Tadpoles are easily tracked when in the middle of a quadrant. (B,C) The image processing algorithms are able to detect tadpoles even when located parallel to the edge of the dish, where they are hard to find by eye, due to the sophisticated background subtraction and healing techniques. The red circle with cross-hairs indicates centroid of the animal shape. (0.21 MB PDF) [file pone.0014370.s003.pdf]

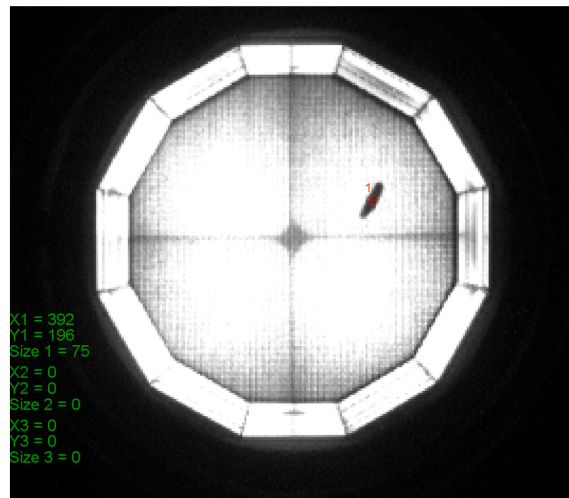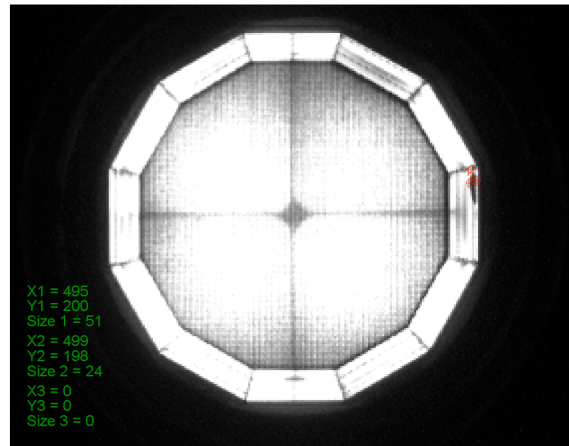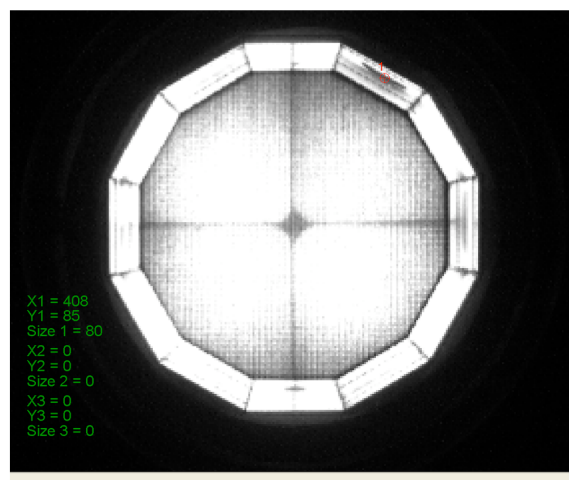

Supplement: Figure S4 — Sample images of planaria within the device. (A) Planaria are easily tracked when in the middle of a quadrant. (B,C) The image processing algorithms are able to detect planaria even when located parallel to the edge of the dish, where they are hard to find by eye, due to the sophisticated background subtraction and healing techniques. The red circle with cross-hairs indicates centroid of the animal shape. (1.21 MB PDF) [file pone.0014370.s004.pdf]
